# Identification of miR-128 Target mRNAs That Are Expressed in B Cells Using a Modified Dual Luciferase Vector

**DOI:** 10.3390/biom13101517

**Published:** 2023-10-13

**Authors:** Sandra Schreiber, Patrick Daum, Heike Danzer, Manuela Hauke, Hans-Martin Jäck, Jürgen Wittmann

**Affiliations:** Division of Molecular Immunology, Department of Internal Medicine III, Nikolaus-Fiebiger-Center of Molecular Medicine (NFZ), Friedrich-Alexander-Universität Erlangen-Nürnberg (FAU), Glückstraße 6, D-91054 Erlangen, Germany

**Keywords:** microRNA, miR-128, dual luciferase assay, HITS-CLIP, miRNA prediction algorithm, B cells

## Abstract

MicroRNAs (miRNAs) are 21–25 nucleotide long non-coding ribonucleic acids that modulate gene expression by degrading transcripts or inhibiting translation. The miRNA miR-128, originally thought to be brain-specific, was later also found in immune cells. To identify a valuable immune cell model system to modulate endogenous miR-128 amounts and to validate predicted miR-128 target mRNAs in B cells, we first investigated miR-128 expression using Northern blot analysis in several cell lines representing different stages of B cell development. The results showed that only primary brain cells showed significant levels of mature miR-128. To study the function of miR-128 in immune cells, we modified dual luciferase vectors to allow easy transfer of 3′ UTR fragments with predicted miR-128 binding sites from widely used single to dual luciferase vectors. Comparison of in silico predicted miR-128-regulated mRNAs in single and dual luciferase constructs yielded similar results, validating the dual luciferase vector for miRNA target analysis. Furthermore, we confirmed miR-128-regulated mRNAs identified in silico and in vivo using the Ago HITS-CLIP technique and known to be expressed in B cells using the dual luciferase assay. In conclusion, this study provides new insights into the expression and function of miR-128 by validating novel target mRNAs expressed in B cells and identifying additional pathways likely controlled by this miRNA in the immune system.

## 1. Introduction

Research on post-transcriptional gene regulation mechanisms has attracted much attention since the discovery of microRNAs (miRNAs), which are gene-regulatory, 21–25 nucleotide (nt) long, single-stranded RNA molecules. The initial primary transcripts, which have the property of forming a hairpin structure, are cleaved in the nucleus by the RNaseIII-like enzyme Drosha [1] together with its cofactor DGCR8 (DiGeorge syndrome critical region gene 8; [2]) into a precursor miRNA. This 60–110 nt stem-loop RNA intermediate is exported to the cytoplasm by the karyopherin Exportin-5 [3,4,5], where it is further processed to the mature 21–25 nt miRNA by Dicer, another RNaseIII-like enzyme [6]. Upon incorporation of the miRNA molecule into the RNA-induced silencing complex (RISC), which consists of one of the four Argonaute (Ago) proteins and additional accessory factors, the double-stranded miRNA molecule is unwound, the passenger strand is degraded, and the mature single-stranded miRNA is loaded into the guide-strand channel of the Ago proteins. In most cases, RISC and the miRNA then interact with the 3′ untranslated region (3′ UTR) of target mRNAs through partial base pairing to induce translational repression and/or mRNA degradation by decapping or deadenylation of the poly(A) tail of the mRNA (reviewed in [7]).

Due to the increase in high-throughput sequencing of small RNAs over the last decades, the number of deposited potential miRNA sequences has steadily increased. Whatever the exact final number, it suggests extensive post-transcriptional regulation of mRNAs by miRNAs. Often, one mRNA is regulated by several miRNAs, and conversely, one miRNA can control hundreds of mRNAs [8,9]. Either way, miRNA regulation of transcripts virtually always leads to lower protein levels.

Identification of miRNA-mRNA target interactions is a critical first step for functional miRNA analysis, followed by experimental validation at a later stage. Commonly used miRNA prediction algorithms include Pictar [8], Targetscan [10], and DIANA-microT web server v.5.0 [11], which still lack sensitivity and specificity despite continuous progress (reviewed in [12]). In addition to these in silico prediction algorithms, experimental methods such as Ago High-throughput sequencing of RNA isolated by cross-linking immunoprecipitation (Ago HITS-CLIP; [13]) have been developed to help reveal those miRNAs with which Ago proteins associate and the likely interaction site of the mRNA with the miRNA in vivo.

Since it is economically not feasible to confirm too many putative miRNA targets by Western blot analyses, heterologous screening with a luciferase assay has proven to be an excellent way to test many predicted target mRNAs in parallel at a relatively low cost. In these assays, the 3′ UTR of interest is cloned downstream of the stop codon of either Renilla or Firefly luciferase, thereby transferring the regulatory capacity of the 3′ UTR to the luciferase reporter gene. The luciferase reporter and a second luciferase expression construct for normalization are transfected into, e.g., the HEK-293 cell line together with miRNA overexpression- or miRNA-sponging constructs [14]. Depending on the co-transfected effector molecule, the target mRNA can be repressed or derepressed via the predicted miRNA target site and the translational output of the reporter can be precisely quantified using a dual luciferase assay (DLA). In addition, the putative miRNA binding site can be mutated, and the loss of miRNA-conferred regulation can be tested (reviewed by [15]).

Initially, miR-128 was thought to be a brain-specific miRNA with weak expression in other organs [16]. Bak and colleagues used microRNA microarray profiling, quantitative reverse transcription PCR (qRT-PCR) analysis, and in situ detection to analyze miRNA expression in the mouse central nervous system in a localized manner. They performed sagittal sections of the adult mouse brain and detected miR-128 accumulation in the neocortex, striatum, hippocampus, thalamus, and granular layer of the cerebellum as well as in the olfactory bulb [17]. A special feature of miR-128, which is encoded by two independent genes, miR-128-1 and miR-128-2, is the regulated processing of their precursors. While miR-128-1 precursor was present in most of the tested 22 normal tissues, mature miR-128-1 was expressed only in the brain and skeletal muscle and at low levels in the cervix and thymus [18]. Northern blot analysis of cell lines representative of some of the tissues has yielded the same results [18]. In 2013, the tissue-specific knockout of both miR-128 paralogs in mouse neurons was reported [19]. The authors showed that miR-128 levels in the striatum bidirectionally regulate motor behavior, while lower levels of miR-128 were associated with greater neuronal excitability and increased motor activity, higher levels suppressed neuronal activity and associated motor behavior. Unexpectedly, several next-generation sequencing approaches of immune cells have identified miR-128 as expressed and differentially regulated miRNA in immune cells [20,21,22]. A study by Nikhat et al. showed that several miRNAs, including miR-128, target myeloid lineage genes in hematopoietic multipotent progenitors (MPPs) and thereby attenuate the myeloid developmental potential. miR-128, among others, serves as an important and non-redundant regulatory component of B and T lymphocyte developmental networks by targeting distinct sets of promiscuously expressed lineage-inappropriate genes to suppress the alternative cell fate options [23]. Yang et al. were the first to demonstrate a role for miR-128 in early B cells. It is abundantly expressed in common lymphoid progenitors (CLPs). Still, it is downregulated in later stages of B cell development, and it inhibits CLPs from developing into progenitor B cells [24]. Yet, the pathways controlled by miR-128 in early B cell development remain unclear.

Dysregulation of miR-128 has been connected to various cancers, including breast, lung, thyroid, head and neck, osteosarcoma, glioma or leukemia, and multiple myeloma (reviewed in [25]). Similarly, miR-128 has also been implicated in mental and behavioral disorders [26], viral infections [27], musculoskeletal disorders [28], or regulation of energy expenditure and metabolic disorders [29], among others. The physiological role of miR-128 in the brain is underlined by the clinical findings of aberrant miR-128 expression in neurodegenerative diseases such as Alzheimer’s disease, Huntington’s disease, or Parkinson’s disease (reviewed in [30]).

Some of the reported roles or controlled pathways or putative mRNA targets of miR-128 are surprising, to say the least, because miR-128 is not expressed at meaningful levels in many of these tissues or cells. Due to miRNA “off-target” interactions, miRNAs with relatively high concentrations in a cell are much more likely to have a biological impact than miRNAs that are present at lower concentrations [31]. Very few Northern blot analyses of endogenous miR-128 levels have been reported, almost all from neuronal tissues or corresponding cells that highly express miR-128 [18,32,33], while Northern blot data from cells that are thought by some to express mature miR-128 are virtually nonexistent. Due to the small number of primary cells, a cell line representing an early B cell stage that endogenously expresses miR-128 would be advantageous. It could be used to analyze in silico predicted miR-128 mRNA targets by knocking down endogenous miR-128 and examining the mRNA and/or protein levels of putative miR-128 targets, complementing the commonly used miRNA overexpression approach. Alternatively, if no suitable B cell line is found, DLAs of predicted miR-128 target mRNAs expressed in B cells could be used to uncover novel miR-128-controlled target mRNAs and pathways in the immune system.

The aim of this study was to gain a deeper understanding of the role of miR-128 in B cells. Identification of B cell lines expressing endogenous miR-128 and validation of predicted target mRNAs expressed in these cells may allow the identification of additional pathways that may be controlled by this miRNA. We have therefore screened several widely used murine and human B cell- and other cell lines for miR-128 expression by Northern blot- and qRT-PCR analysis and customized dual luciferase reporter vectors for miRNA target analysis, which we validated and used to characterize in silico and in vivo identified novel miR-128 mRNA targets known to be expressed in B cells.

## 2. Materials and Methods

### 2.1. Animals

Male C57BL/6 mice [34], 8–10 weeks old, from Charles River (Sulzfeld, Germany) were maintained under specific pathogen-free conditions in the animal unit of the Nikolaus-Fiebiger-Center (Erlangen, Germany).

### 2.2. Cells, Cell Lines and Cell Culture Conditions

The cell lines used in this study are summarized in Table 1. A brain cell suspension from C57BL/6 mice was prepared by forcing two brains through a plastic mash into a 6-well plate (Greiner Bio-One, Frickenhausen, Germany) filled with PBS (Thermo Fisher Scientific, Dreieich, Germany), washing the cell suspension once in PBS before lysing the cells directly in peqGOLD TriFast reagent (peqlab, Erlangen, Germany) for RNA isolation. The human cervical carcinoma cell line HeLa (ATCC Cat. No. CCL-2; [35]), the murine pro-B cell line 38B9 [36], the murine immature B cell line WEHI-231 (ATCC Cat. No. CRL-1702; [37]), the murine plasma B cell line Ag8H (a subclone of X63-Ag8.653 described in [38]) and the mature splenic B cell line CH27 [39] were cultured in RPMI 1640 medium supplemented with 10% fetal bovine serum, 1% L-glutamine, 1% sodium pyruvate, 1% penicillin/streptomycin, 0.1% β-mercaptoethanol (all from Thermo Fisher Scientific) at 37 °C and 5% CO_2_ in a humidified incubator. The human plasma B cell line INA-6 [40] was cultured in RPMI 1640 medium supplemented with 20% fetal bovine serum, 1% L-glutamine, 1% sodium pyruvate, 1% penicillin/streptomycin, 0.1% β-mercaptoethanol and 2 ng/mL recombinant human IL-6 (Strathmann Biotec AG, Hamburg, Germany). The murine fibroblast cell line NIH3T3 (ATCC Cat. No. CRL-1658; [41]), the human embryonic kidney cell line HEK-293 (ATCC Cat. No. CRL-1573) [42], the human glioblastoma cell line A172 (ATCC Cat. No. CRL-1620) [43] and the murine neuroblastoma cell line Neuro-2a (ATCC Cat. No. CCL-131) [44] were grown in DMEM medium supplemented with 10% fetal bovine serum, 1% L-glutamine, and 1% penicillin/streptomycin (all from Thermo Fisher Scientific) and maintained at 37 °C and 7.5% CO_2_ in a humidified incubator.

### 2.3. Cloning of Fragments with Predicted miR-128 Binding Sites for Luciferase Assays

Several 3′ UTR fragments and an exonic sequence with predicted miR-128 binding sites were identified via Targetscan [11], Pictar [12], DIANA-microT web server v.5.0 [13] or Ago HITS-CLIP data [13] (Table 2). They were amplified from genomic DNA or cDNA of the murine B cell line WEHI-231 using specific primer pairs (see Table 3) and the FastStart High Fidelity PCR System (Roche Applied Science, Mannheim, Germany). After cloning the PCR products into the pCR2.1 vector (Thermo Fisher Scientific) and sequencing, the correct 3′ UTR fragments were inserted into the single luciferase vector pRL-TK (Promega, Mannheim, Germany, Cat. No. E2241) or the dual luciferase vector psiCHECK-4 (this work) by restriction digest cloning (all restriction enzymes from NEB, Frankfurt, Germany). Correct clones were confirmed by control digests and sequencing (Seqlab, Göttingen, Germany).

### 2.4. Construction of miRNA Expression Vectors

Fragments of approximately 500 bp containing the precursors of miR-128-2 and miR-201 were amplified from genomic DNA of the murine B cell line WEHI-231 using the FastStart High Fidelity PCR System (Roche) and the primers listed in Table 4. After cloning the PCR products into the pCR2.1 vector and sequencing, the correct miRNA cassettes were inserted into the mammalian expression vector pcDNA3 (Thermo Fisher Scientific) by restriction digest cloning. Restriction digests and sequencing confirmed correct clones.

### 2.5. Construction of psiCHECK-3 and psiCHECK-4 Vectors

To obtain restriction enzyme-compatible dual luciferase vectors for pRL-TK (Promega), the psiCHECK-2 vector (Promega, Cat. No. C8021) was modified as follows: first, the vector was cleaved at the *Xba*I site downstream of the Firefly luciferase gene and filled-in by treatment with the Klenow fragment of DNA Polymerase I (NEB) to destroy the single *Xba*I site, resulting in psiCHECK-2 w/o *Xba*I (not reported here). To construct psiCHECK-3, pBluescript II KS(+) (Stratagene, Heidelberg, Germany, Cat. No. 212207) was digested with *Not*I/*Xho*I, the 70 bp MCS was purified from a 2.0% agarose gel using the QIAEXII Gel Extraction Kit (QIAGEN, Hilden, Germany) and cloned into the *Not*I/*Xho*I sites of psiCHECK-2 w/o *Xba*I. To construct psiCHECK-4 with *Xba*I/*Not*I cloning sites, the oligonucleotides MCS psiCHECK-4 for and MCS psiCHECK-4 rev (Table 4) were annealed and cloned into the *Not*I/*Xho*I sites of psiCHECK-2 w/o *Xba*I. Correct clones were identified by restriction digests and confirmed by sequencing for all constructs.

### 2.6. Northern Blot Analysis

RNA samples were isolated from the indicated cell lines or primary cells using peqGOLD TriFast reagent (peqlab) and quantified on a NanoDrop ND-1000 spectrometer (peqlab). 20 µg total RNA together with 2.5 µg GeneRuler Ultra Low Range DNA Ladder (Thermo Fisher Scientific, Cat. No. SM1211) were separated on a 10% Urea Polyacrylamide gel after 1 h pre-run in 1 × TBE buffer until the bromophenol blue running front had almost run out of the gel. The gel was stained with ethidium bromide to check for equal loading and RNA quality and then transferred onto a nylon membrane (GeneScreen Plus, PerkinElmer, Zaventems, Belgium) by semi-dry electroblotting (Sedec M system, peqlab) in 1 × TBE buffer. The RNA was cross-linked to the wet membrane in a UV-crosslinker (Stratalinker, Stratagene) and subsequently baked at 80 °C for 1 h. The marker bands were visualized under UV light and marked with a pencil on the nylon membrane.

The baked nylon membrane was pre-hybridized with hybridization solution (5× SSC, 20 mM Na_2_HPO_4_ pH 7.2, 7% SDS, 2× Denhardts solution, 40 µg/mL denatured salmon sperm DNA) at 45 °C in a hybridization oven (Hybaid, Fisher Scientific, Schwerte, Germany) for at least 3 h. Labeling of 20 pmol of miR-16 DNA oligo (Table 4) or miR-128 LNA oligo (Exiqon, Vedbaek, Denmark) was performed with T4 polynucleotide kinase (NEB) and 50 µCi [γ-^32^P] ATP (10 mCi/mL) (Hartmann Analytic, Braunschweig, Germany) at 37 °C for 1 h. After heat inactivation of the kinase for 10 min at 68 °C, the probe was purified using a G-25 Sephadex Quick Spin Column (Roche Applied Science), and the incorporation rate was determined using a scintillation counter (Tri-Carb 2800TR, PerkinElmer, Rodgau, Germany). The pre-hybridization solution was replaced with a new, pre-warmed hybridization solution containing the labeled probe and hybridized overnight at 45 °C in a hybridization oven. After discarding the hybridization solution, the membrane was washed in washing solution (3× SSC, 25 mM NaH_2_PO_4_ pH 7.5, 5% SDS, 10× Denhardts solution) at 45 °C in a hybridization oven twice for 10 min and twice for 30 min. The membrane was wrapped in Saran wrap and exposed to a phosphoimager plate (BAS MP2040, Fuji, Tokyo, Japan) overnight. Detection was performed using a Phosphoimager (FLA-3000, Raytest, Straubenhardt, Germany) and BASReader and AIDA Image Analysis software (version 4.26), respectively.

The membrane was stripped with 80 mL 1% SDS at 85 °C for 30 min under constant rotation in a hybridization oven. Overnight exposure to a phosphoimager plate confirmed the successful removal of the hybridization signal.

### 2.7. Transfection of HEK-293 Cells and Luciferase Assays

Briefly, 1 × 10^5^ HEK-293 cells were seeded one day before transfection in 24-well plates (Greiner Bio-One) in 1 mL DMEM medium containing 10% fetal bovine serum without antibiotics. The next day, 7.5 ng of dual luciferase plasmid or 100 ng of each of the single luciferase vectors pRL-TK and pGL3 control (Promega, Cat. No. E1741) were transfected together with 600 ng of empty control vector or miRNA expression vector using Lipofectamine 2000 (Thermo Fisher Scientific). Luciferase assays were performed 48h after transfection using the Dual-Luciferase reporter system (Promega, Cat. No. E1980) on a Sirius single tube luminometer (Berthold Technologies, Bad Wildbad, Germany) according to the manufacturer’s instructions. Briefly, DMEM medium was gently removed from the cells, and 100 µL of 1× passive lysis buffer (Promega, Cat. No. E1941) was added to the cells and incubated at RT for 15 min on an orbital shaker. 100 µL of Luciferase assay reagent II (LAR II) was added to appropriate luminometer assay tubes (Greiner Bio-One), and 20 µL of cell lysate was transferred to the tube containing LAR II and mixed by pipetting 2 or 3 times. After quantification of the Firefly luminescence, this reaction is quenched, and the Renilla luciferase reaction is simultaneously initiated by adding 100 µL of Stop and Glo reagent to the same tube. The Firefly and Renilla luminescence values of HEK-293 cells transfected with an empty control vector were subtracted from the experimental values as background. All experiments were performed in triplicate and repeated at least three times. Significance was tested using the Mann–Whitney test for unpaired data (Prism 7.0, GraphPad Software, Boston, MA, USA). *p* values < 0.05 were considered significant.

### 2.8. Quantification of miRNAs by TaqMan Quantitative RT-PCR Analysis

One day before transfection, 1 × 10^5^ HEK-293 cells were seeded into 24-well plates in 1 mL DMEM medium containing 10% fetal bovine serum without antibiotics. The next day, 600 ng of empty pcDNA3 control vector, miR-128- or miR-201 expression vectors were transfected using Lipofectamine 2000, and total RNA including miRNAs was isolated 48h later using the miRNeasy Mini kit (QIAGEN, Cat. No. 217004). Briefly, the cell layer was directly lysed by adding QIAzol lysis reagent (700 μL final volume), vortexed for 1 min, and incubated at RT for 5 min before further processing following the manufacturer’s instructions. RNA concentrations and purity (absorption at 260 nm and a ratio of 260/280 of ~2.0, respectively) were determined using NanoDrop.

Isolated miRNAs were first transcribed into cDNAs using the TaqMan MicroRNA Reverse Transcription Kit (Thermo Fisher Scientific, Cat. No. 4366597). Briefly, 5 μL RNA (2 ng/μg) and 3 μL of 5× primer stocks [Thermo Fisher Scientific, miR-128-3p (Cat. No. 002216), miR-201-5p (Cat. No. 002578) or RNU6B (Cat. No. 001093)] were added to 7 μL master mix (0.15 μL dNTP, 1 μL reverse transcriptase, 1.5 μL buffer, 0.2 μL RNase inhibitor, and 4.15 μL RNAase-free water). Mixtures were incubated in a GeneAmp PCR 9700 Thermocycler (Thermo Fisher Scientific) for 30 min at 16 °C, 30 min at 42 °C, and 5 min at 85 °C. The cDNA preparation was then pre-diluted 1:5 with nuclease-free water and quantified using the TaqMan Universal Master Mix II (Thermo Fisher Scientific, Cat. No. 4427788). Each sample was assayed in triplicate. For each reaction, 5 μL cDNA (1:5), 0.75 μL miRNA-specific probe mix (20×), 7.5 μL master mix, and 1.75 μL nuclease-free water were mixed in 96-well plates (Thermo Fisher Scientific, Cat. No. AB-1100) and covered with adhesive qPCR Plate Seals (Thermo Fisher Scientific, Cat. No. AB-1170). TaqMan qRT-PCR analysis was performed on an Applied Biosystems 7300 Real-Time PCR System (Thermo Fisher Scientific). Reactions of cDNAs prepared without reverse transcriptase (RT-minus) and without the cDNA template (NTC) served as negative controls to validate the specificity of the reaction. The mean of the Ct-values (cycle threshold) was calculated for the triplicates of each sample. The mean Ct of the housekeeping gene RNU6B was subtracted from the mean Ct of each miRNA for normalization to equal input, and relative expression was calculated using the comparative Ct method. Significance was tested using the Mann–Whitney test for unpaired data (Prism 7.0, GraphPad Software, Boston, MA, USA). *p* values < 0.05 were considered significant.

## 3. Results

### 3.1. Northern Blot Analysis Shows That Only Primary Brain Cells Express Mature miR-128

**Figure 1:**
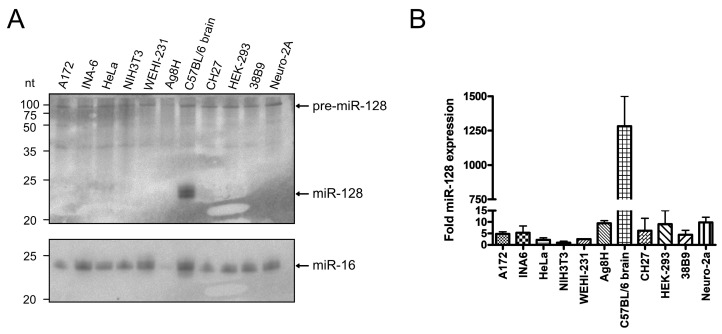
Analysis of mature miR-128 levels in different mouse- and human cell lines and primary cells. Total RNA was isolated from the indicated cell lines and mouse brain and used for (**A**) Northern blot- and (**B**) quantitative reverse transcription PCR (qRT-PCR) analysis. (**A**) 20 µg of total RNA was separated in a 10% Urea/Polyacrylamide gel and subjected to Northern blot analysis using radioactively labeled oligo probes for miR-128 (upper panel) and miR-16 (lower panel) as loading control. Radioactive signals were detected by phosphorimaging. (**B**) The same RNA as in (**A**) was used for Taqman qRT-PCR analysis of miR-128 and RNU6B levels. Relative miR-128 expression was calculated by normalizing input RNA levels to RNU6B and arbitrarily setting the value for NIH3T3 cells to 1. Data are presented as the standard error of the mean (SEM) of two independent experiments. Original images of (A) can be found in Figure S1.

We decided to use Northern blot analysis to screen several cell lines representing different B cell stages for endogenous miR-128 expression to establish an in vitro model to study miR-128 function in B cells. In addition, we included widely used murine and human cell lines as well as primary cells from the brain of C57BL/6 mice as a positive control ([45], Table 1). After isolation of total RNA and separation in a Urea/Polyacrylamide gel, Northern blot analysis was performed, and the membrane was probed with a radiolabeled, LNA-modified miR-128 probe (Figure 1A, upper panel). While a signal for the potential pre-miR-128 can be detected at about 90 nt for all cell lines, only the mouse brain showed a signal for mature miR-128 at around 22/23 nt. The weak band at approximately 40-nt could represent the unspecific binding of an RNA degradation product or to an unknown processed form of miR-128. Loading of similar RNA amounts was evaluated with a radiolabeled miR-16 probe (Figure 1A, lower panel). To assess miR-128 abundance by a second, independent method and to better quantify miR-128 levels, RNA was also analyzed by Taqman qRT-PCR analysis (Figure 1B). The miR-128 levels were normalized for equal RNA input by subtracting the RNU6B levels, and the miR-128 value for NIH3T3 cells, which expressed the lowest miR-128 amount of the cell lines tested, was arbitrarily set to 1. While the amount of mature miR-128 was barely detectable in all cell lines tested, miR-128 levels in the brain were more than 1200-fold higher than in NIH3T3 cells. Consistent with the Northern blot results, brain-derived cells such as A172 or Neuro-2a did not show a signal for mature miR-128, possibly indicating that cells with reduced or absent miR-128 expression levels are selected during in vitro culture. In conclusion, miR-128 expression could only be detected by Northern blot analysis in primary cells isolated directly from mouse brains and not in several murine and human B cell lines or other cell lines tested.

### 3.2. Generation of Compatible Dual Luciferase Vectors to Facilitate the Transfer of Fragments from Single Luciferase Vectors

**Figure 2:**
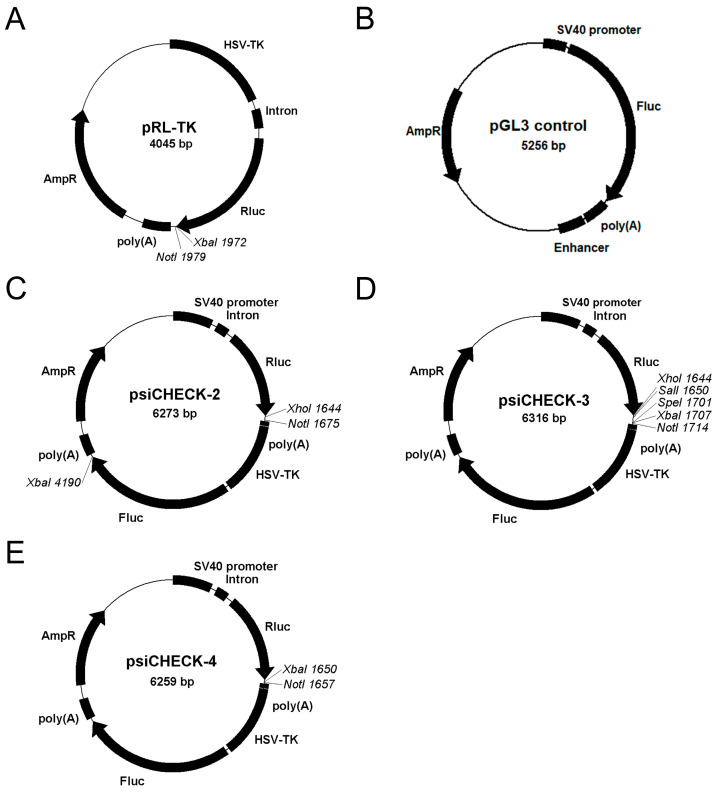
Schematic representation of the single- and dual-luciferase vectors used and generated in this study. Schematic representation of the single luciferase vectors (**A**) pRL-TK, (**B**) pGL3 control, and the dual luciferase vectors (**C**) psiCHECK-2, (**D**) psiCHECK-3, and (**E**) psiCHECK-4. HSV-TK: Herpes simplex virus thymidine kinase gene promoter; SV40 promoter: Simian virus 40 promoters; Intron: chimeric intron; Rluc: Renilla luciferase, Fluc: Firefly luciferase, poly(A): polyadenylation signal, Enhancer: Simian virus 40 enhancer; AmpR: Ampicillin resistance gene. Recognition sites of singular restriction enzymes for cloning are indicated.

To circumvent the lack of a miR-128-expressing B cell line and still be able to validate predicted possible miR-128-controlled target mRNAs expressed in B cells, we resorted to performing luciferase reporter assays. One of the most commonly used commercial vectors is Promega’s pRL-TK, which uses the Renilla luciferase reporter gene. (Figure 2A). Recognition sites located downstream of the Renilla stop codon allow cloning of the 3′ UTR of the mRNA of interest at this position to verify possible regulation of Renilla luciferase mRNA by miRNAs. Typically, a second luciferase vector encoding Firefly luciferase, such as the vector pGL3 control, is co-transfected as a normalization control (Figure 2B). To analyze miRNA regulation of 3′ UTR fragments, cells must be transfected with two luciferase plasmids plus either miRNA-expression- or sponging vectors or chemical miRNA mimics/inhibitors. This approach was simplified by combining both luciferase genes as independent transcription units on one plasmid in the commercially available vector psiCHECK-2 (Figure 2C), thus minimizing calculation or pipetting errors. However, a disadvantage of this vector is that the small multiple cloning site is not compatible with widely used luciferase plasmids such as pRL-TK or other luciferase vectors, making the transfer of 3′ UTR fragments by cloning not straightforward. To change this, we modified the psiCHECK-2 vector by first mutating the *Xba*I site downstream of the firefly luciferase gene. If more single restriction sites are required, the multiple cloning site of the pBluescript II vector was inserted into the modified psiCHECK-2 vector, resulting in the vector psiCHECK-3 with five single restriction sites (*Xho*I, *Sal*I, *Spe*I, *Xba*I, and *Not*I) downstream of the Renilla luciferase gene (Figure 2D). When restriction sites compatible with the pRL-TK vector are required, an annealed double-stranded oligonucleotide with *Xba*I and *Not*I restriction sites was cloned into the modified psiCHECK-2 vector, yielding the vector psiCHECK-4 (Figure 2E). This modification also minimizes the number of additional nucleotides that could act as artificial recognition sites for miRNAs.

### 3.3. Comparison of Single vs. Dual Luciferase Constructs for miRNA Target Analysis

**Figure 3:**
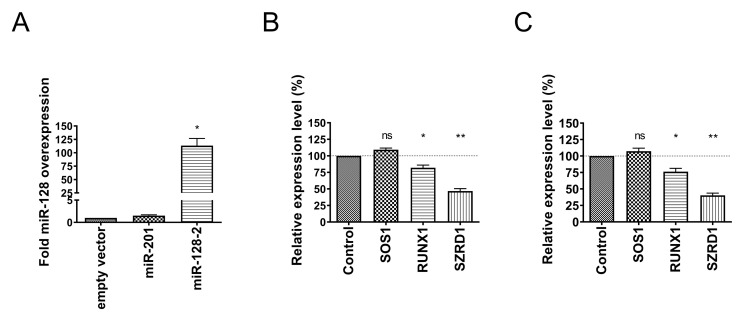
Validation of miR-128 expression vectors and comparison of single- versus dual luciferase assays. (**A**) HEK-293 cells were transfected in triplicate with empty, control miR-201 or miR-128 expression vectors. After 48 h, total RNA was isolated and reverse transcribed using specific RT primers for miR-128 or RNU6B as a loading control. Quantitative real-time PCR analysis using specific Taqman probes was performed independently three times in triplicate. miR-128 Ct values were normalized to RNU6B Ct values, and the ΔΔCt value of cells transfected with the empty vector control was arbitrarily set to 1. Data are presented as the standard error of the mean (SEM). * *p* value < 0.05. (**B**) HEK-293 cells were transfected in triplicate with the single Renilla luciferase vector pRL-TK containing the indicated 3′ UTR inserts, the Firefly luciferase vector pGL3 control for normalization of transfection efficiency, and an empty expression vector (control) or a miR-128 expression vector. After 48 h, protein extracts were prepared and used for dual luciferase assays. The Firefly and Renilla luciferase activity ratio was calculated and arbitrarily set to 100% for the control vector transfections. Data are presented as the SEM of at least three independent experiments performed in triplicate. * *p* value < 0.05, ** *p* value < 0.01, ns: not significant. (**C**) HEK-293 cells were transfected in triplicate with the dual luciferase vector psiCHECK-4 containing the indicated 3′ UTR inserts as in (**B**) and an empty expression vector (control) or a miR-128 expression vector. After 48 h, protein extracts were prepared and used for dual luciferase assays. The Firefly and Renilla luciferase activity ratio was calculated and arbitrarily set to 100% for the control vector transfections. Data are presented as the SEM of at least three independent experiments performed in triplicate. Significance was tested using the Mann–Whitney test for unpaired data. * *p* value < 0.05, ** *p* value < 0.01, ns: not significant.

To test whether experiments using the adapted dual luciferase vector psiCHECK-4 would yield the same results as a single luciferase approach, side-by-side pilot experiments were performed. For the overexpression of miR-128, an expression vector based on the commonly used pcDNA3 plasmid was first created and tested. The empty vector control pcDNA3 was used to determine whether the transfection reagent or the transfection process itself had any cytotoxic effects on the HEK-293 cells. The unrelated miRNA miR-201 was cloned and used as a negative control to ensure that the potential upregulation of miR-128 is specifically associated with the miR-128 expression cassette. qRT-PCR analysis revealed that transfection of HEK-293 cells with the miR-128 expression construct alone resulted in an approximately 113-fold overexpression of miR-128 compared to the empty vector control. Overexpression of miR-201 did not result in increased amounts of miR-128 (Figure 3A). This allows the miR-128 expression vector to be used in single/dual luciferase assay validation studies.

The miRNA prediction algorithms Targetscan, Pictar, and DIANA microT were used to identify mRNAs with predicted miR-128 binding sites and potential roles in B cell development (Table 2). These were then analyzed for expression in B cells using expression data from the Immunological Genome Project (ImmGen), which contains microarray- and RNAseq gene expression data for nearly all immune cells [46].

Based on these criteria, one of the selected mRNAs is SOS1 [son of sevenless homolog 1 (Drosophila)], a guanine nucleotide exchange factor for RAS proteins ([47]; Table 5). RUNX1 (runt-related transcription factor 1) is a subunit of a transcription factor that binds to the core element of many enhancers and promoters and is known to be involved in the development of normal hematopoiesis (reviewed in [48]). SZRD1 [SUZ RNA Binding Domain Containing 1, also known as C1ORF144 (chromosome 1 open reading frame 144) or PM18/PM20/PM22 (Putative MAPK-Activating Protein)] is an understudied protein with the primary function of inhibiting cell proliferation and inducing cell apoptosis [49].

All three 3′ UTR fragments were cloned into pRL-TK- and psiCHECK-4 vectors, and HEK-293 cells were transfected with either two single luciferase vectors (pRL-TK with Renilla luciferase and the respective 3′ UTR and the pGL3 Firefly luciferase control) or the dual luciferase vector psiCHECK-4 and the respective 3′ UTR. In both cases, the empty pcDNA3 vector, the miR-128 expression construct, or the miR-201 expression vector as an unrelated control vector were added. Each transfection was performed in triplicate, and cells were lysed 48 h after transfection. Transfection of the empty luciferase vectors and subtraction of their luciferase activities allowed determination and elimination of background. After calculating the ratio of Renilla- to Firefly luciferase activity to control for variation in DNA concentrations, cell number, and transfection efficiency, values obtained with the empty control vector were set to 100%. In all cases, luciferase values obtained with the miR-201 expression vector behaved similarly to the empty control vector and are therefore not included in the graph. Overexpression of miR-128 together with the SOS1 3′ UTR fragment did not result in a significant change in luciferase activity in either the single or dual-luciferase approach, indicating that this 3′ UTR is not a target of miR-128 in heterologous luciferase reporter assays (compare Figure 3B,C). The RUNX1 3′ UTR analysis by single- and dual-luciferase assays revealed significantly reduced Renilla luciferase activity in both cases, suggesting that miR-128 may target RUNX1. Finally, testing of the 3′ UTR of SZRD1 resulted in a significant decrease in Renilla luciferase activity in both approaches, indicating that SZRD1 contains functional miR-128 binding sites. In conclusion, the results obtained by comparing both the single and dual luciferase approaches yielded very comparable results, validating the use of the dual luciferase vector for the study of predicted miRNA target sites.

### 3.4. Verification of In Vivo Identified miR-128 Binding Sites by the Dual Luciferase Assay

In our small-scale verification experiments for the DLA, two of the three in silico predicted miR-128-regulated mRNAs showed significant regulation. We hypothesized that the data generated by the Ago HITS-CLIP technique should be enriched for true miRNA target mRNAs and that the number of experimentally confirmed miRNA target mRNAs from this approach should be higher than that of the in silico predictions.

**Figure 4:**
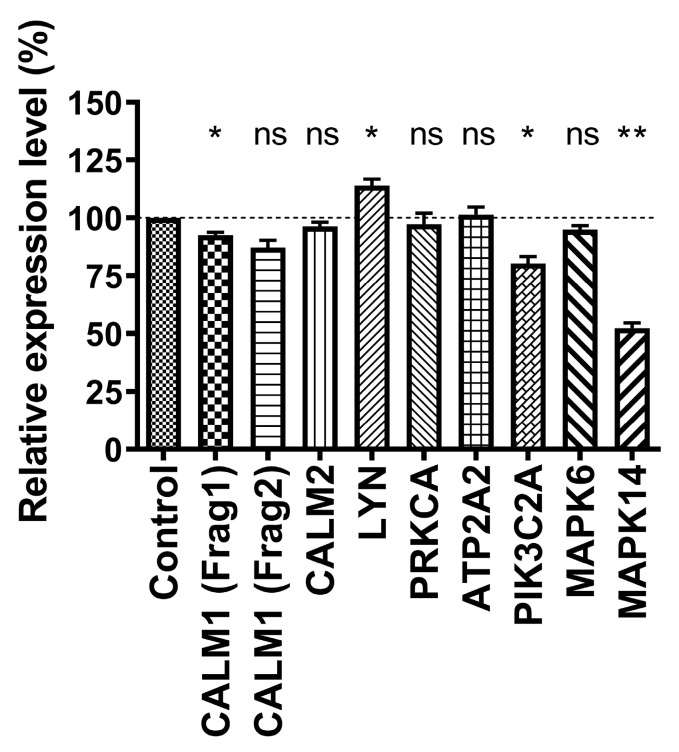
Verification of in vivo identified miR-128 target mRNAs by dual luciferase assays. HEK-293 cells were transfected in triplicate with the dual luciferase vector psiCHECK-4 containing the indicated 3′ UTR inserts or exonic sequences (PRKCA) and an empty expression vector (control) or a miR-128 expression vector. After 48 h, protein extracts were prepared and used for dual luciferase assays. The Firefly and Renilla luciferase activity ratio was calculated and arbitrarily set to 100% for the control vector transfections. Data are presented as the standard error of the mean (SEM) of at least three independent experiments performed in triplicate. Significance was tested using the Mann–Whitney test for unpaired data. * *p* value < 0.05, ** *p* value < 0.01, ns: not significant.

Since we did not find any B cell Ago HITS-CLIP dataset to evaluate, we used published mouse brain data [13], as miR-128 is also highly expressed there. Within identified Ago-mRNA clusters, putative Ago binding sites were analyzed for co-incident 6–8 nucleotide sequence motifs representing possible miR-128 binding sites. Potential target mRNAs were screened for co-expression in B cells using the ImmGen database and included Calmodulin 1, Calmodulin 2, Lyn, PRKCA, ATP2A2, PIK3C2A, and the MAP kinases (MAPK) MAPK6 and MAPK14 (Table 2). A comparison of these in vivo identified Ago-bound transcripts with the results of target prediction algorithms revealed that only a small fraction of them were also identified as putative miR-128 targets in silico (Table 5). For one of the Ago HITS-CLIP target mRNAs, PRKCA, a miR-128 binding site was detected in exon 4 of the coding region. Since most miRNA target algorithms only predict binding sites in the 3′ UTRs, no miR-128 prediction was available for PRKCA. Fragments of the selected mRNAs were cloned into psiCHECK-4 vectors and analyzed by DLAs as described above. Four of the eight Ago HITS-CLIP identified mRNAs with putative Ago cross-linking- and predicted miR-128 binding sites were found to be putatively regulated by miR-128 in dual luciferase reporter assays (Figure 4).

The success rates were mixed when comparing which of the identified in vivo binding sites were also listed in three of the most common miRNA prediction algorithms (Targetscan, Pictar, DIANA microT). While several non-predicted targets such as Calmodulin 2, PRKCA, and ATP2A2 also did not show miR-128 regulation in the DLA, non-predicted targets such as PIK3C2A and LYN showed significant regulation (with LYN amounts being surprisingly slightly increased rather than decreased upon miR-128 expression). Calmodulin 1 was only predicted by DIANA microT and showed significant regulation, while MAPK6, predicted by Targetscan and DIANA microT, did not show a significant regulation. Only MAPK14, which has two miR-128 binding sites predicted by all three algorithms, showed a strong down-regulation upon miR-128 overexpression in HEK-293 cells. Taken together, these data indicate that mRNAs with experimentally identified in vivo Ago binding sites and 6–8 nt nucleotide sequence motifs representing possible miR-128 binding sites do not automatically outperform bioinformatic predictions as determined in limited heterologous luciferase reporter assays.

## 4. Discussion

The initial goal of this study was to identify a murine or human B cell line that endogenously expresses miR-128 to further our understanding of this understudied microRNA in the immune system. In addition to overexpressing miR-128 in this cell line, complementary miR-128 sponging experiments would also be possible to elucidate miR-128 regulated targets and pathways in B cells. Validation studies often use miRNA sponges (sometimes called miRNA inhibitors, antagomirs [50], or miRNA sponges [14]), which only makes sense if the miRNA of interest is present in reasonable amounts in the cells of interest. One example is the widely used colorectal cancer-derived cell line HCT116. Bai and colleagues used HCT116 cells to test whether FOXO4 would be a target for miR-128 in a miRNA sponge approach [51]. However, miR-128 is only present at trace levels in HCT116 cells [31]. Even if the miRNA of interest is expressed in the organ/cell line used, many studies have failed to test whether the predicted mRNA targets are actually expressed there as well. Additionally, of course, if the miRNA of interest is to be overexpressed from a plasmid construct, it should be determined whether the processing of the miRNA works and how much the miRNA is overexpressed.

Our finding that only primary brain cells and not murine and human B cell lines showed signals for mature miR-128 is consistent with the observation that miR-128 is predominantly expressed in the brain [16] or in brain-derived tumors [52,53,54]. The finding that the miR-128 precursor is present in all cell lines analyzed may indicate that transcription and processing of the miR-128 gene(s) to pre-miR-128 levels occurs in all cells but that the final maturation step from the stem-loop-structured precursor to mature miR-128 is regulated. Lee and colleagues also reported similar findings on miR-128 levels [18]. Either appropriate processing proteins are present to allow the processing of pre-miR-128 to its mature form, as in brain cells, or inhibitory factors are present in all other cells analyzed except the brain, inhibiting the further processing of pre-miR-128. Factors such as RNA-binding proteins that regulate miRNA biogenesis could be involved [55], or RNA-binding proteins could inhibit the access of miRNAs to their target sequence in the 3′ UTR, as has been described for Dnd1 [56]. Another, although unlikely, possibility could be different stabilities of mature miR-128 in other cell lines or tissues. Since hematopoietic MPPs and CLPs are scarce primary cell populations and are not available in large quantities for experimentation, further attempts will be necessary to find an established lymphoid cell line that can be used to study endogenous miR-128 function.

A DLA approach was pursued as an alternative experimental attempt to narrow down the miR-128-controlled pathways in B cells. Initial validation studies of three in silico predicted potential miR-128 mRNA targets known to be expressed in B cells in single versus dual luciferase reporter assays in HEK-293 cells showed very similar results, qualifying the use of the modified dual luciferase vectors for mRNA target analysis. The compatibility with the commonly used single luciferase vector pRL-TK and the simplified transfection procedure minimizes a potential source of variability (transfection of two plasmids instead of three). In addition, transfection of less DNA (7.5 ng vs. 200 ng plasmid DNA in a 24-well format) means less cell stress and probably less non-specific activity. Two of the three predicted mRNA targets selected showed significant regulation by miR-128 in HEK-293 cells (see below).

The Ago HITS-CLIP methodology promised to yield more authentic and relevant miRNA target mRNAs than in silico prediction algorithms because the data is derived from cells or tissues in vivo [13]. Since no Ago HITS-CLIP data on B cells were available, we resorted to brain-derived, Ago-bound mRNAs with existing miR-128 seed sites. We selected eight RNAs with known expression and roles in B cell development for further analysis in the heterologous DLAs in HEK-293 cells. This analysis revealed that four of the eight mRNAs showed considerable regulation in HEK-293 cells. In particular, mRNA targets also identified by in silico target prediction programs (CALM1, MAPK14) also showed significant regulation, while most other in vivo identified mRNAs did not show a significant down-regulation. Interestingly, analysis of the 3′ UTR of the kinase Lyn even showed a significant upregulation in the DLA. That miRNAs can lead to mRNA up-regulation under certain circumstances has already been described [57].

It cannot be excluded that not all mRNAs identified in vivo could be verified in vitro due to the heterologous HEK-293 system used in this study. Although they may best mimic the in vivo situation in DLAs, B-lymphoid cell lines cannot be used due to the generally very low transfection efficiencies. HEK-293 cells are the most commonly used heterologous cell system in DLAs because they are robust, widely available, and easy to transfect. Caveats, such as their origin as an embryonic kidney cell line and the potential absence or presence of factors in HEK-293 cells, must be considered. Ideally, 3′ UTRs, as long as possible, should be used in DLAs to mimic the natural situation as closely as possible. Unfortunately, only oligonucleotides with minimal miRNA binding sites are often used, which do not reflect all characteristics of the endogenous 3′ UTR. Several other explanations are also possible. For example, the anti-Ago antibody 2A8 used for Ago HITS-CLIP in Chi et al. also recognizes Radixin and could explain some non-specific binding results [58]. Equally surprising is the fact that virtually no miR-128 was detected in the Ago brain cell immunoprecipitations and NGS analysis using the 2A8 antibody reported in this manuscript, which is in contrast to brain miRNA frequencies as measured by cloning frequencies or bead-based flow cytometric miRNA expression profiling in the human brain [59]. Similarly, Ago HITS-CLIP data generated with the 2A8 Ago antibody identified mRNAs with miR-128 seed matches only at rank 79 in the AGO-miRNA CLIP data [13]. However, this may also be due to general problems with the CLIP technique, including, amongst others, the unclear efficiency of Argonaute protein cross-linking, the preferential targeting of uridines, or the uncertainty of which small RNA guided the interaction of the RNA-binding protein with individual target mRNAs.

It is essential to identify only functionally relevant microRNA targets in a sea of computationally predicted target mRNAs. This critical issue is often insufficiently addressed in miRNA studies, leading to overconfidence or overinterpretation of molecular mechanisms explaining, e.g., phenotypes of genetically engineered organisms. Unfortunately, a substantial proportion of retracted papers fabricated by paper mills involve miRNAs, and certainly, hundreds or perhaps thousands of published papers involving miRNAs remain unidentified [31]. This dramatically complicates attempts to use literature searches to distinguish fabricated research from papers containing legitimate findings—putative target mRNAs of miR-128 are, unfortunately, no exception. It would be ideal to study miRNAs in the correct cellular context and, if not applicable, as seems to be the case for miR-128 in cell lines, at least to investigate whether the miRNA of interest and the putative mRNA controlled by this miRNA are actually expressed in the cell type or cell line of interest. An essential tool in this direction for immunological questions is the comprehensive ImmGen database. If heterologous assays need to be performed in HEK-293 cells, ideally, full-length 3′ UTRs should be used rather than isolated minimal miRNA response elements, potentially allowing mutagenesis studies of binding sites in these reporter mRNAs. Potential caveats of high-throughput CLIP-based data should also be considered. Confirmation of predicted miR-128 target genes at the protein level by Western blot analysis from tissues or cells with endogenous miR-128 expression is particularly valuable and rare, such as published miR-128-driven changes in Szdr1, Pea15a, Arpp21, or pERK2 amounts [19] or MALT1 and A2B amounts in CLPs [24].

This study has identified and confirmed six new miR-128-regulated mRNAs expressed in B cells. While the biological role of slightly increased LYN amounts after miR-128 expression remains to be investigated in more detail, the data suggest that miR-128 in B cells may play a role in phosphoinositide 3-kinase (PI3K) signaling pathways involved in cell proliferation, oncogenic transformation, cell survival, cell migration, and intracellular protein trafficking through regulation of PIK3C2A. One of the pathways of which some members are known to be regulated by miR-128 is the MAP kinase (MAPK) signaling pathway [19,24]. We confirmed miR-128 regulation of SZRD1 (PM18/PM20/PM22, [60]), a member of this pathway, and added the putative miR-128-controlled MAPK14. This serine/threonine kinase is an essential component as one of the four p38 MAPKs that play an important role in the cascade of cellular responses triggered by extracellular stimuli such as proinflammatory cytokines or physical stress, leading to direct activation of transcription factors. One of these transcription factors could be the possibly miR-128-controlled RUNX1, which is involved in generating hematopoietic stem cells and their differentiation into myeloid and lymphoid lineages [48]. CALM1 (Calmodulin 1) is one of three calmodulin proteins that are members of the EF-hand calcium-binding protein family, which act as part of a calcium signal transduction pathway by mediating the control of a large number of enzymes, ion channels, aquaporins, and other proteins via calcium-binding. The intracellular abundance of calcium ions is a crucial triggering signal during the establishment of a B cell response to the antigen (reviewed in [61]).

## 5. Conclusions

In conclusion, we have shown that none of the murine and human B cell lines tested expressed endogenous levels of miR-128. This is in contrast to early primary murine bone marrow B cells, where miR-128 was well detected by Northern blot- and qRT-PCR analysis (Schreiber et al., unpublished). To gain insight into miR-128 regulatory cascades in immune cells, we modified and validated a simplified dual luciferase vector for several miR-128 target mRNAs predicted in silico or identified in vivo using Ago HITS-CLIP analysis. Six miR-128 target mRNAs with robust expression in B cells were identified, which will need to be confirmed at the protein level in B lymphoid cells. Their associated pathways are promising for further investigation related to miR-128 expression in early B cells.

## Figures and Tables

**Table 1:** Cell lines used for Northern blot- and qRT-PCR analysis.

Cell Line	Cell Properties	Citation
C57BL/6	murine primary cells from brain	[34]
HeLa	human cervical carcinoma cell line	[35]
38B9	murine pro-B cell line	[36]
WEHI-231	murine immature B cell line	[37]
Ag8H	murine plasma B cell line, subclone of X63-Ag8.653	[38]
CH27	murine mature splenic B cell line	[39]
INA-6	human plasma B cell line	[40]
NIH3T3	murine fibroblast cell line	[41]
HEK-293	human embryonic kidney cell line	[42]
A172	human glioblastoma cell line	[43]
Neuro-2A	murine neuroblastoma cell line	[44]

**Table 2:** Predicted miR-128 target mRNAs and fragments thereof tested in this study.

Gene Symbol	Refseq ID	3′ UTR nts	Exon
SZRD1	NM_001025608	36–925	---
RUNX1	NM_001111021	2747–3746	---
SOS1	NM_009231	16–1812	---
CALM1 (Frag1)	NM_009790	777–1780	---
CALM1 (Frag2)	NM_009790	2382–3376	---
CALM2	NM_007589	5–624	---
LYN	NM_001111096	388–1403	---
PRKCA	NM_011101	---	Exon 4
ATP2A2	NM_001110140	828–1808	---
PIK3C2A	NM_011083	3–888	---
MAPK6	NM_015806	110–1120	---
MAPK14	NM_011951	1–580	---

**Table 3:** Primers used for amplification of 3′ UTRs.

Gene Symbol	Primer for (5′-3′)	Primer rev (5′-3′)	Product (bp)
SZRD1	TCTAGACGCCTCCTGGATGGTCTG	GCGGCCGCGCCTTTCTGAGCCCAGAGTC	903
RUNX1	TCTAGACATCGCCATCGAGGGACT	GCGGCCGCAAAGCATGCCAGAATGAGGA	1015
SOS1	TCTAGAGATAGTTTCCTAGCCCCCAGA	GCGGCCGCCTTTGCATCCAAGAAGGGATT	1810
CALM1 (Frag1)	TCTAGAATTGTACAGAATGTGTTAAATTTCTTG	GCGGCCGCGAGCAACAATTGGGTAAATTGTAA	1017
CALM1 (Frag2)	TCTAGAGTCAAGGCAGTACCCCTGAG	GCGGCCGCGGCTGCAGAAATGTTTATTGAA	1008
CALM2	TCTAGAATTGTACAGAATGTGTTAAATTTCTTG	GCGGCCGCGAGCAACAATTGGGTAAATTGTAA	633
LYN	ACTAGTTTACAGGTGAAGCCACAAGC	GCGGCCGCTCAATCCCTTCGCCTAGTCA	1029
PRKCA	TCTAGAGACCCCAGGAGCAAGCAC	GCGGCCGCTGTCACATTTCATCCCTTGG	126
ATP2A2	TCTAGATGTCTTGTTCTTAATGGCCTTG	GCGGCCGCAGCTGGCTGCACACCTAAAC	994
PIK3C2A	TCTAGATTCCGACTTCTGAGCTTTGG	GCGGCCGCCCTGAGCACGTTACCAAACA	899
MAPK6	TCTAGATGCAAGGATTTTTCTTGGTTC	GCGGCCGC GCCTAGGGATGGGGAATAGA	1024
MAPK14	TCTAGAGCACCTGGTTTCTGTTCTGTC	GCGGCCGCAACTATCTACGCGCCCTTCTC	594

**Table 4:** Primer used for amplification of miRNA expression constructs, for the generation of the multiple cloning site, and as Northern blot probes.

Plasmid	Primer for (5′-3′)	Primer rev (5′-3′)	Product (bp)
Mmu-miR-128-2	GTTAACCTCGAGGCCCAAGTGCTTTGTTGTTT	TGATCATCTAGAATCGATCGCAGCAGGAGCTCTTTAGT	478
Mmu-miR-201	AAGCTTCCACCTCAATACTGACCCTAGAA	GATATCCGATATATCTTGCCTTCCACTTG	488
MCS psiCHECK-4	TCGACTCTAGAGC	GGCCGCTCTAGAG	---
Mmu-miR-128 probe	Exiqon, proprietary sequence	---	---
Mmu-miR-16 probe	CGCCAATATTTACGTGCTGCTA	---	---

**Table 5:** Prediction of miR-128 target mRNAs by different algorithms.

Gene Symbol	Targetscan (Sites)	Context Score	Pictar (Sites)	Score (Mm)	DIANA microT (Sites)	miTG Score
SZRD1	4	−0.98	6	21.8503	4	51.44
RUNX1	2	−0.24	not found	---	2	9.00
SOS1	4	−0.23	2	6.6930	4	16.54
CALM1 (Frag1)	0	---	0	---	1	7.33
CALM1 (Frag2)	0	---	0	---	1	7.33
CALM2	0	---	0	---	0	---
LYN	0	---	not found	---	0	---
PRKCA	n.a. ^1^	---	n.a. ^1^	---	n.a. ^1^	---
ATP2A2	0	---	not found	---	0	---
PIK3C2A	0	---	0	---	0	---
MAPK6	1	−0.22	0	---	1	11
MAPK14	2	−0.77	2	4.6117	2	28.11

^1^ n.a. not applicable.

## Data Availability

The data presented in this study are available on request from the corresponding author.

## Supplementary Materials

The following supporting information can be downloaded at: https://www.mdpi.com/article/10.3390/biom13101517/s1, Figure S1: Original images of Figure 1A.
