# Supplementary figures and images for: Identification of miR-128 Target mRNAs That Are Expressed in B Cells Using a Modified Dual Luciferase Vector
Source: Biomolecules. 2023 Oct 13;13(10):1517. doi: 10.3390/biom13101517 (PMC10605364; doi:10.3390/biom13101517)

Figure S1

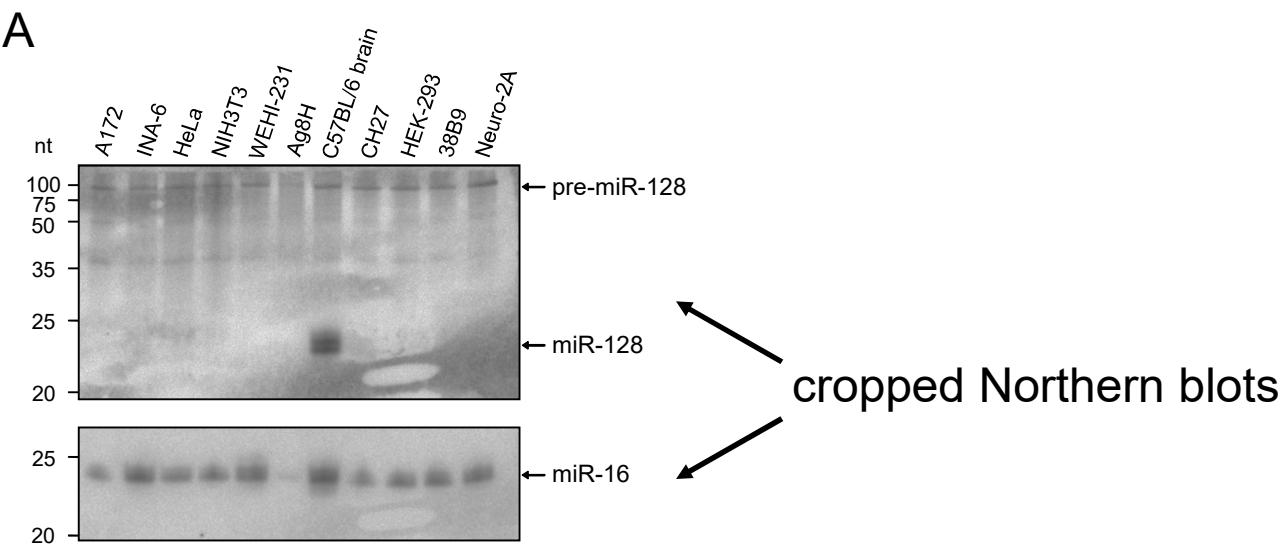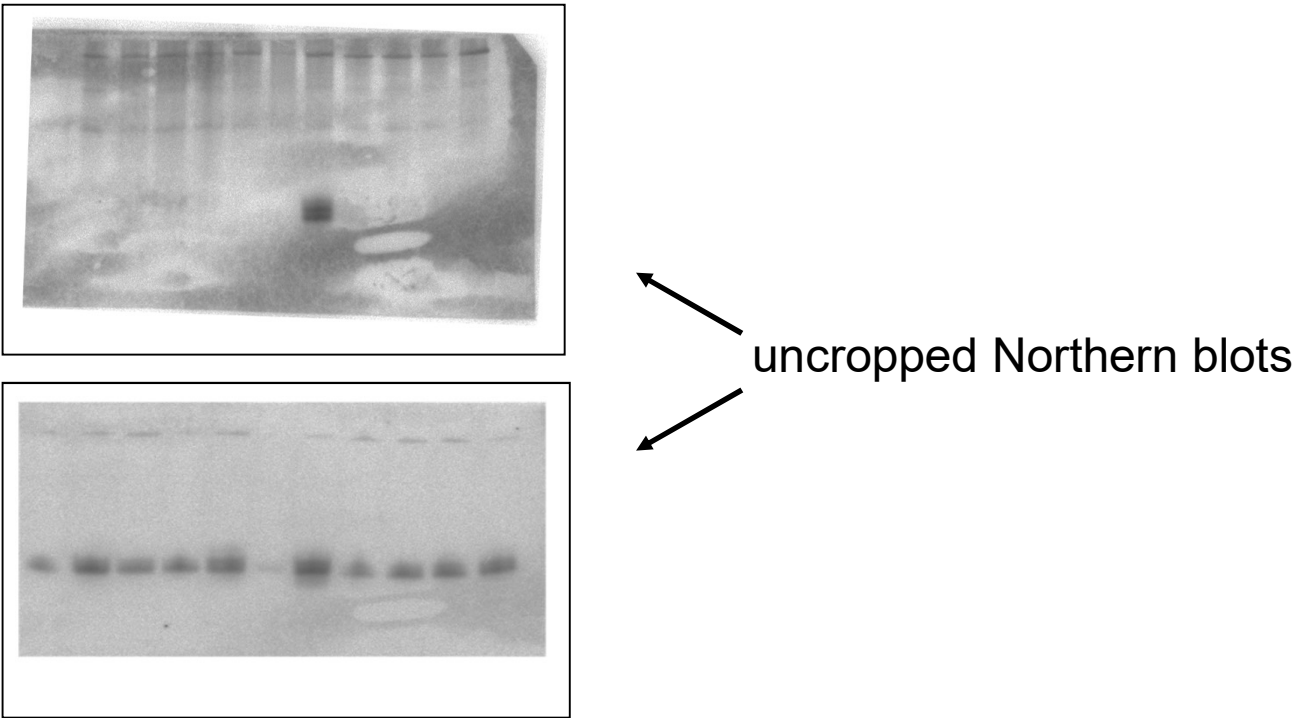

Supplement: Supplementary file 1 [file biomolecules-13-01517-s001.zip › biomolecules-2622608-supplementary.pdf]
